# Utilizing Monte Carlo Simulations to Optimize Institutional Empiric Antipseudomonal Therapy

**DOI:** 10.3390/antibiotics4040643

**Published:** 2015-12-11

**Authors:** Sarah J. Tennant, Donna R. Burgess, Jeffrey M. Rybak, Craig A. Martin, David S. Burgess

**Affiliations:** 1 Pharmacy Services, University of Kentucky HealthCare, 800 Rose Street, H110, Lexington, KY 40536, USA; E-Mails: sarah.tennant@uky.edu (S.J.T.); donna.burgess@uky.edu (D.R.B.); jrybak@uthsc.edu (J.M.R.); 2 College of Pharmacy, University of Kentucky, Biological Pharmaceutical Building, 789 S. Limestone Street, Lexington, KY 40536, USA; E-Mail: david.burgess@uky.edu; 3 College of Graduate Health Sciences, University of Tennessee, 920 Madison Avenue, Suite 407, Memphis, TN 38163, USA

**Keywords:** antimicrobial stewardship, pharmacodynamics, *Pseudomonas aeruginosa*, pharmacokinetics, modeling

## Abstract

*Pseudomonas aeruginosa* is a common pathogen implicated in nosocomial infections with increasing resistance to a limited arsenal of antibiotics. Monte Carlo simulation provides antimicrobial stewardship teams with an additional tool to guide empiric therapy. We modeled empiric therapies with antipseudomonal β-lactam antibiotic regimens to determine which were most likely to achieve probability of target attainment (PTA) of ≥90%. Microbiological data for *P. aeruginosa* was reviewed for 2012. Antibiotics modeled for intermittent and prolonged infusion were aztreonam, cefepime, meropenem, and piperacillin/tazobactam. Using minimum inhibitory concentrations (MICs) from institution-specific isolates, and pharmacokinetic and pharmacodynamic parameters from previously published studies, a 10,000-subject Monte Carlo simulation was performed for each regimen to determine PTA. MICs from 272 isolates were included in this analysis. No intermittent infusion regimens achieved PTA ≥90%. Prolonged infusions of cefepime 2000 mg Q8 h, meropenem 1000 mg Q8 h, and meropenem 2000 mg Q8 h demonstrated PTA of 93%, 92%, and 100%, respectively. Prolonged infusions of piperacillin/tazobactam 4.5 g Q6 h and aztreonam 2 g Q8 h failed to achieved PTA ≥90% but demonstrated PTA of 81% and 73%, respectively. Standard doses of β-lactam antibiotics as intermittent infusion did not achieve 90% PTA against *P. aeruginosa* isolated at our institution; however, some prolonged infusions were able to achieve these targets.

## 1. Introduction

*Pseudomonas aeruginosa* is a ubiquitous Gram negative organism that has been implicated as the causative pathogen in many nosocomial infections. According to the National Healthcare Safety Network, *P. aeruginosa* is the fifth most common cause of hospital-acquired infections [1]. Mortality due to *P. aeruginosa* infection is high with some studies _ENREF_3estimating mortality rate around 40% [2,3].

Antimicrobial stewardship programs (ASPs) must provide guidance to clinicians to help influence therapy selection and antimicrobial utilization. Successfully doing so will optimize both patient outcomes and healthcare costs, and curtail the development of antimicrobial resistance [4]. Infections due to *P. aeruginosa* are particularly challenging as designing effective antimicrobial regimens is hampered by growing resistance to a limited number of active agents. Consequences of ineffective antimicrobial therapy are increased mortality and costs of care [1,5,6,7]. One tool that ASPs can use to guide antipseudomonal therapy is the institutional antibiogram: the collection of quantitative minimum inhibitory concentrations (MIC) and reporting of qualitative susceptibility results for the microbial isolates at a given institution [8]. However, antibiograms only provide the likelihood that a pathogen will be susceptible to a given antimicrobial agent as defined by regulatory bodies based on historical data. The selected agent must be administered at appropriate doses to optimize antimicrobial pharmacokinetic and pharmacodynamic parameters [9].

Monte Carlo simulations can be used by ASPs as an extension of the antibiogram to guide optimal dosing of antimicrobials. A Monte Carlo simulation is a mathematical model developed in the 1940s to simulate scenarios that require the generation of random numbers. It has many applications in physics, finance and business, artificial intelligence, and video game design. In the setting of antimicrobial therapeutics, Monte Carlo simulations can combine pharmacokinetic and microbiological data to predict the likelihood an antimicrobial regimen will achieve a therapeutic target [10]. This is called the probability of target attainment (PTA) where the target to be achieved is an optimal pharmacodynamic parameter for bacterial killing [11]. This study aims to determine which empiric beta-lactam antimicrobial regimens will achieve a PTA of at least 90% against *P. aeruginosa* isolates at our institution.

## 2. Methods

### 2.1. Data Collection

This was a single-center, retrospective analysis conducted at University of Kentucky Chandler Medical Center, a 718-bed academic medical center. This study was approved by the Institutional Review Board. Clinical microbiology laboratory data was obtained for *P. aeruginosa* isolates collected between 1 January 2012 and 31 December 2012. Samples included for analysis were isolated from patients ≥18 years old who were admitted as inpatients during the study period. The first positive isolate from any culture site per year for *P. aeruginosa* from each patient was included for analysis. Subsequent positive cultures were excluded as recommended by antibiogram guidelines [8]. Culture sources included blood, bone, intra-abdominal, respiratory, skin/wound, urine, and miscellaneous sites. Data collected included source of isolate, patient location within hospital, and minimum inhibitory concentration (MIC) for formulary anti-pseudomonal beta-lactam antibiotics. At the time of study, MICs were reported using the BD Phoenix^®^ automated microbiology system. The anti-pseudomonal beta-lactam antibiotics on formulary include aztreonam, cefepime, meropenem, and piperacillin/tazobactam.

A PubMed search of the primary literature was conducted *a priori* to identify pharmacokinetic parameters for incorporation into the pharmacodynamic model. Search terms included the name of the agent, “healthy”, “volunteer”, and “pharmacokinetics”. Pharmacokinetic parameters collected from the identified studies included total body clearance (Cl_TB_), volume of distribution (V_d_), and half-life (t_1/2_). Protein binding (PB) was obtained from the manufacturers’ package inserts.

### 2.2. Model Construction

A 10,000 trial Monte Carlo simulation was constructed for each antimicrobial regimen in Oracle^®^ Crystal Ball for Microsoft Excel^®^ (version 11.1.2, Redwood City, CA, USA). Commonly prescribed doses were analyzed as both intermittent infusion (30 min) and prolonged infusion (3 h) for each antibiotic. The pharmacodynamic target used was free time above MIC (*f*%T > MIC). The optimal *f*%T > MIC used for carbapenems was 40% of the dosing interval, for cephalosporins and aztreonam was 70%, and for penicillins was 50%. These values have correlated with bacterial killing, and reduced mortality *in vivo* [9].

Intermittent infusion [12]:

(1) $$f\text{\%T }>\text{MIC}=\text{ln}\left\{\left[\text{dose }\times \;\left(1-\text{PB}\right)\right]÷\left({\text{V}}_{\text{d}}\;\times \text{MIC}\right)\right\}\;\times \left({\text{V}}_{\text{d}}÷{\text{Cl}}_{\text{TB}}\right)\times \left(100÷\tau \right)$$

Prolonged infusion [13]:

(2) $$f\text{\%T}>\text{MIC}=\left\{{\text{T}}_{\text{inf}}-\;\left\{\ln\left[\left({\text{R}}_{0}÷{\text{Cl}}_{\text{TB}}\right)÷\left({\text{R}}_{0}\;÷{\text{Cl}}_{\text{TB}}-\text{MIC}\right)\right]\times \left({\text{t}}_{1/2}÷0.693\right)\right\}\right\}+\;\left\{\left[\text{ln}\left({\text{R}}_{0}÷{\text{Cl}}_{\text{TB}}\right)-\text{ln}\left(\text{MIC}\right)\right]\times \left({\text{t}}_{1/2}÷0.693\right)\right\}\times \left(\frac{100}{\tau }\right)$$

(3) $${\text{R}}_{0}=\left[\text{dose }\times \;\left(1-\text{PB}\right)\right]/{\text{T}}_{\text{inf}},\;{\text{T}}_{\text{inf}}=\text{infusion time},\;\tau =\text{dosing interval}$$

The model identifies a pharmacokinetic parameter that falls within a lognormal distribution of the standard deviation about the mean and incorporates that into each simulation. Each simulation incorporated an MIC from the distribution of MICs identified from microbiologic data in order to mimic practice where clinicians do not know the MIC of the organism upon initiation of empiric antimicrobial therapy.

## 3. Results

Two hundred seventy-two *P. aeruginosa* isolates were identified for inclusion. Sixty-one percent of isolates were from male patients and 39% were from females. Forty-seven percent of specimens were isolated from patients admitted to a surgical or medical intensive care unit, 40% were isolated from patients admitted to an acute care service, 8% were admitted to our institution’s oncology wing, and location was unknown in 4% of cases. The most common sources were respiratory (42%), skin/wound (24%), urine (19%), blood (8%), and miscellaneous sites (5%).

Table 1 indicates the distribution of MICs reported for study antibiotics and the percent of susceptible isolates according to 2012 Clinical and Laboratory Standards Institute (CLSI) standards [14]. The MIC_50_, and MIC_90_ of *P. aeruginosa* isolates are also presented in Table 1. Cefepime (81%) had the highest susceptibility rate against *P. aeruginosa*.

**Table 1:** (Minimum inhibitory concentrations) MIC Range, MIC_50_, and MIC_90_, and percent susceptible against *P. aeruginosa* isolates from University of Kentucky.

	Breakpoint ^a^ (mcg/mL)	MIC Range (mcg/mL)	MIC_50_ (mcg/mL)	MIC_90_ (mcg/mL)	% Susceptible
Aztreonam	8	≤2–≥32	8	32	68
Cefepime	8	≤1–≥32	4	16	81
Meropenem	2	≤1–≥16	1	8	74
Piperacillin	16	≤2–≥128	8	128	75

MIC = minimum inhibitory concentration, mcg/mL; MIC_50_ = MIC value at which growth was inhibited in 50% of isolates; MIC_90_ = MIC values at which growth was inhibited in 90% of isolates; ^a^ According to 2012 CLSI guidelines [14].

**Figure 1:**
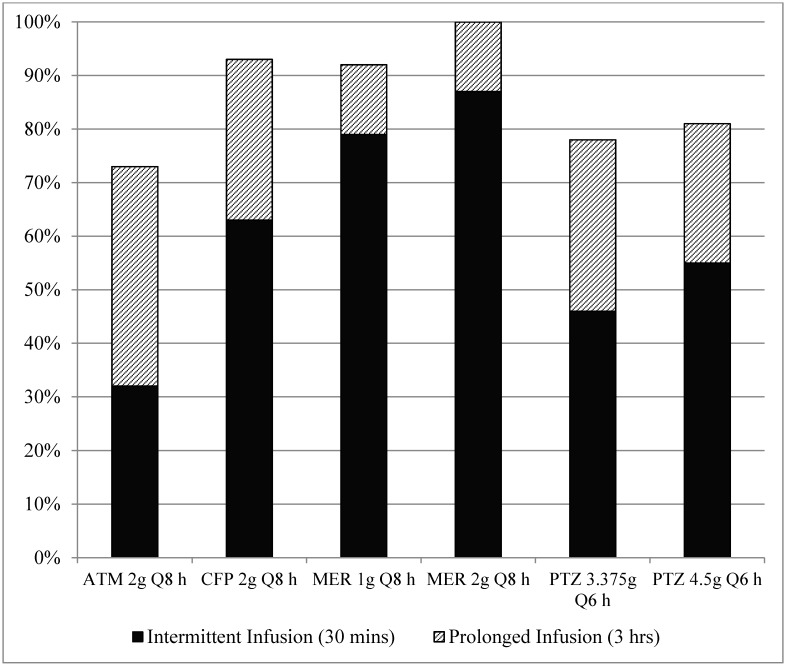
Probability of target attainment of optimized empiric antipseudomonal beta-lactams. ATM = aztreonam; CFP = cefepime; MER = meropenem; PTZ = piperacillin/tazobactam.

Identified pharmacokinetic studies and parameters included in the Monte Carlo simulation model are listed in Table 2. Data is presented as mean values and standard deviation. In one study of meropenem pharmacokinetics, no standard deviation was provided for t_1/2_, so a variation of 10% was set in the model [15]. Figure 1 presents the PTA for empiric antimicrobial regimens across the range of MICs encountered at our institution. Intermittent infusions of beta-lactams over 30 min did not reach pharmacodynamic targets in 90% of simulations. Prolonged infusions of cefepime 2000 mg every 8 h, meropenem 1000 mg every 8 h, and meropenem 2000 mg every 8 h have 93%, 92%, and 100% probability of reaching pharmacodynamic targets, respectively.

**Table 2:** Pharmacokinetic parameters incorporated into model.

Antimicrobial Agent	Clearance (L/h)	Volume of Distribution (L)	Half Life (h)	Protein Binding (%)
Aztreonam [16,17]	5.45 ± 1.24	13.7 ± 4.94	1.69 ± 0.43	56
Cefepime [18,19]	8.58 ± 1.5	18.4 ± 3.8	2.32 ± 0.39	20
Meropenem [15,20]	11.28 ± 1.86	12.5 ± 1.5	0.98	2
Piperacillin [21,22]	11.07 ± 2.59	11.2 ± 2.1	0.7 ± 0.11	30

## 4. Discussion

*Pseudomonas aeruginosa* is a major pathogen implicated in nosocomial infections. Successful treatment of *P. aeruginosa* is difficult due to limited antimicrobial options and increasing drug resistance. Providing guidance for the treatment of *P. aeruginosa* is particularly challenging for antimicrobial stewardship practitioners who must balance using effective antimicrobials, preserving the utility of these agents and managing healthcare costs. Antipseudomonal treatment success is not only dependent on appropriate antimicrobial agent choice but also on optimal dosing to achieve pharmacodynamic targets. We used institutional MIC data and healthy volunteer pharmacokinetic data to model pharmacodynamic target attainment of available empiric β-lactam regimens used when *P. aeruginosa* is suspected.

Results of this study show that intermittent infusions of meropenem and prolonged infusions of meropenem or cefepime are most likely to achieve PTA >90% against *P. aeruginosa* isolates at our institution. Interestingly, prolonged infusion regimens of piperacillin/tazobactam were not able to reach PTA >90%, even at high doses of 4000 mg every 6 h. This may be attributable to high MICs of piperacillin/tazobactam against *P. aeruginosa* in our population. In the range of MICs encountered against *P. aeruginosa* in this study, the highest MICs were for piperacillin/tazobactam. Eight percent of isolates had an MIC of 64 mcg/mL and 10% had an MIC of 128 mcg/mL. The tested antimicrobial regimens are more likely to reach PTA against isolates expressing lower MICs and less likely to reach these targets when the MIC is at the higher end of the range. Additionally, in patients with normal renal function—as modeled in this study—it is unfeasible to reach and maintain therapeutic serum concentrations above the MIC for 50% of the dosing interval when the MICs are elevated.

The current study is not the only example of using institution-specific isolates in Monte Carlo simulations to influence antimicrobial dosing practices. Goff *et al.* analyzed 64 *P. aeruginosa* isolates from their institution and conducted a Monte Carlo Simulation to determine PTA for carbapenems and cefepime. Cefepime 2000 mg every 8 h administered over 0.5–1 h achieved a PTA of 86% while infusion over 3–4 h achieved a PTA greater than 90%. The antimicrobial stewardship team at their institution decided to change empiric cefepime dosing to prolonged infusion with resultant reductions in length of stay in both the hospital and the ICU, 14-day mortality, and in-hospital mortality [23].

Another study evaluated implementation of a clinical pathway for antimicrobial therapy in ventilator-associated pneumonia (VAP). A clinical pathway was designed using Monte Carlo simulation results from MICs against *P. aeruginosa* isolated from respiratory sources in three intensive care units between November 2004 and July 2005. Based on Monte Carlo simulations, cefepime 2000 mg prolonged infusion every 8 h, meropenem 2000 mg prolonged infusion every 8 h, and piperacillin/tazobactam 4.5 g prolonged infusion every 6 h or 18 g continuous infusion every 24 h had the highest PTA against *P. aeruginosa* in the population of ICU patients with VAP. After implementing this clinical pathway, patients had decreased infection-related mortality, improved time to appropriate antimicrobial therapy, and decreased infection-related length of stay [24].

This study is limited in that it is a retrospective review of microbiological data and makes predictions based on mathematical modeling. While it reflects current guidelines regarding construction of an institutional antibiogram by including data from one institution, other institutions may have differing results [25]. Antimicrobial stewards must consider their institutional microbiome and local susceptibility patterns when making empiric therapy decisions. Future application includes using the Monte Carlo methodology with unit-specific isolates as CLSI encourages stratification of cumulative antibiogram data by nursing unit or site of care [25].

Our model was based on population pharmacokinetics from normal weight, healthy volunteers. In our patient population, 47% were located in an intensive care unit, creating the potential for confounding. We chose healthy volunteer population due to homogeneity and consistency of data throughout the published pharmacokinetic literature. In a study by Lodise *et al.* that conducted Monte Carlo simulations using pharmacokinetic parameters simulated from hospitalized patients and collected from healthy subject studies, the healthy subject studies underestimated PTA [26]. Therefore, the results of our study likely reflect worst-case, lower PTA than what would be achieved clinically. These results should be applied cautiously for patients with alterations in clearance or volume of distribution. Additionally, our model is built around predicted serum concentrations of the tested antimicrobials. Future models for specific sites of infection should incorporate tissue penetration to calculate PTA.

The PTA goals in our model were conservative and represent optimal pharmacodynamic outcomes to maximize bacterial killing *in vitro*, but there is a paucity in the current body of literature to support clinical outcomes associated with targeting these optimal pharmacodynamic targets using Monte Carlo simulation, and available published studies are conflicting [9]. One study conducted by Fish *et al.* compared outcomes predicted by Monte Carlo simulation with actual clinical outcomes in 182 critically ill patients with *P. aeruginosa* pneumonia [27]. Both modeling and direct estimation were used to ascertain pharmacodynamic targets. There was no correlation between actual clinical response to therapy and Monte Carlo simulation predicted target attainment.

These studies and the current study set the stage for future direction of the application of Monte Carlo simulation in ASPs. They can be used as an extension of the antibiogram, inform institutional clinical pathway design, and influence physicians to choose the correct agent, dose, route, and dosing interval. These are important metrics of antimicrobial use processes that can be evaluated by ASPs to track and optimize antimicrobial utilization [28]. Since the execution of the current study, the antimicrobial management team provides practitioners at our institution with Monte Carlo simulation data in addition to the annual antibiogram to help guide empiric therapy for both *P. aeruginosa* and Enterobacteriaceae. Future applications include building Monte Carlo models to evaluate the dosing regimens of new antipseudomonal agents. Ceftolozane-tazobactam and ceftazidime-avibactam were recently approved for the treatment of complicated intra-abdominal infection or complicated urinary tract infection [29,30,31,32]. Monte Carlo simulations utilizing pharmacokinetics of these agents in patients combined with local isolates can provide direction for clinicians on use in more difficult to treat infections such as pneumonia and bacteremia. Additionally, currently utilized dosing schemes can be evaluated against clinical isolates as these agents begin to be used in practice. Around 3% of tested *P. aeruginosa* demonstrated resistant MICs with these new agents which may require higher doses, shorter intervals, and/or prolonged infusions to achieve pharmacodynamic targets and bactericidal activity [33,34]. Antimicrobial stewardship teams must ensure that these new agents are utilized appropriately and dosed optimally to preserve activity against *P. aeruginosa*.

## 5. Conclusions

ASPs can use Monte Carlo simulations as another tool in addition to the antibiogram to determine optimal empiric therapy regimens. Using local microbiology data and pharmacokinetic data, ASPs can develop unit-specific or institution-wide empiric regimens to target *P. aeruginosa*. Manipulating dosing and administration modalities can achieve optimal pharmacodynamic targets to improve the likelihood of successfully treating an infection. At our institution, prolonged infusions of high dose cefepime and meropenem achieved pharmacodynamics targets against *P. aeruginosa*. There are opportunities for further studies to examine the clinical application of Monte Carlo simulations in designing empiric antimicrobial therapy.
